# P-74. Characteristics and Clinical Outcomes of Lyme Arthritis: A Single-Center, Retrospective Study

**DOI:** 10.1093/ofid/ofaf695.303

**Published:** 2026-01-11

**Authors:** Khalid Abu-Zeinah, Sofia Molina Garcia, Madiha Fida, Omar M Abu Saleh

**Affiliations:** Mayo Clinic Rochester, Rochester, MN; Mayo Clinic, Rochester, Minnesota; Mayo Clinic, Rochester, Minnesota; Mayo Clinic, Rochester, Minnesota

## Abstract

**Background:**

Lyme arthritis is a manifestation of disseminated infection with Borrelia spp., typically presenting as knee monoarthritis weeks to months after infection. Most patients respond to antibiotics, but some develop antibiotic-refractory Lyme arthritis (ARLA), characterized by proliferative or autoimmune synovitis despite clearance of active infection, with proposed risk factors including treatment delay, host genetics, and intra-articular steroid use prior to antibiotics.

**Table 1:**
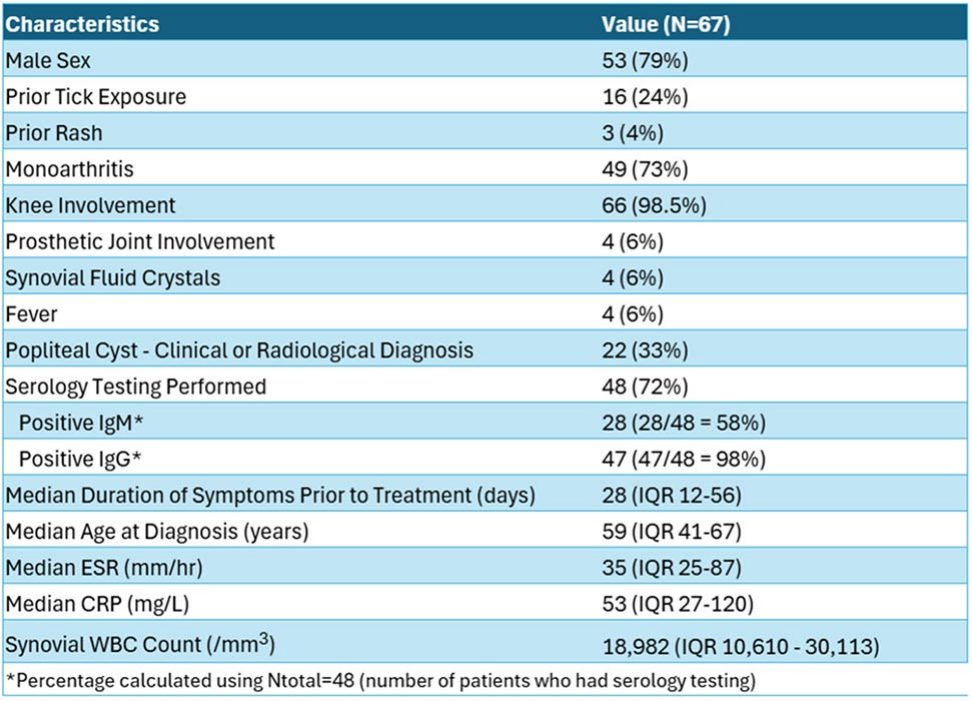
Characteristics of cohort. IQR = interquartile range, ESR = erythrocyte sedimentation rate, CRP = C-reactive protein, WBC = white blood cell.

**Table 2:**
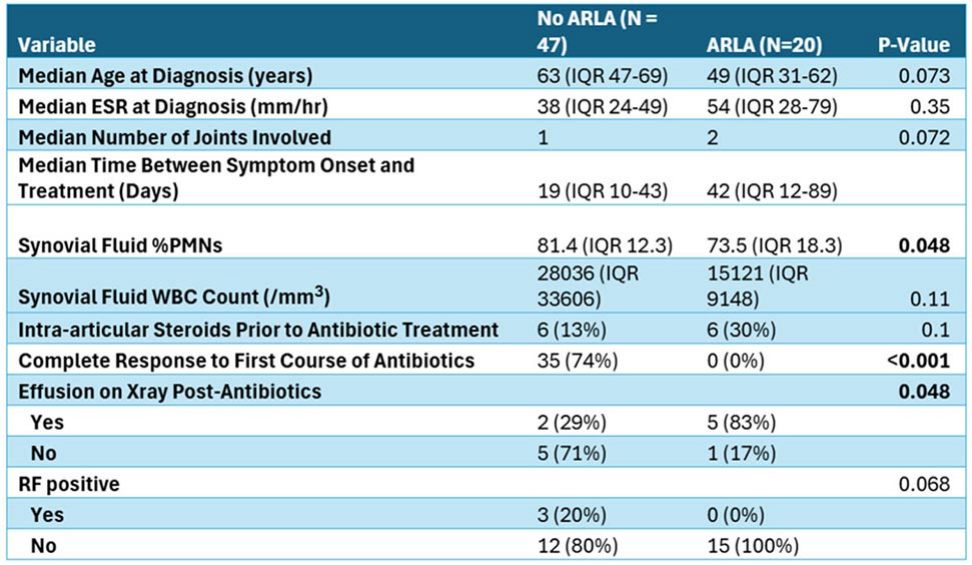
Comparison of characteristics and outcomes between ARLA and non-ARLA patients

**Methods:**

We performed a retrospective chart review of adult patients with Lyme PCR-positive synovial fluid at our center between 01/2004 and 02/2025.  ARLA was defined as persistent inflammatory arthritis despite ≥8 weeks of antibiotics and/or negative repeat PCR after antibiotics. Statistical analysis was done on R and STATA.

**Figure 1:**
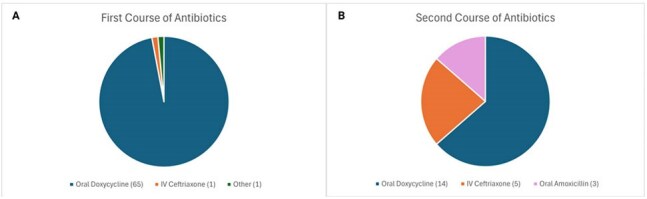
Type of antibiotics administered during the first course (A) and the second course (B)

**Figure 2:**
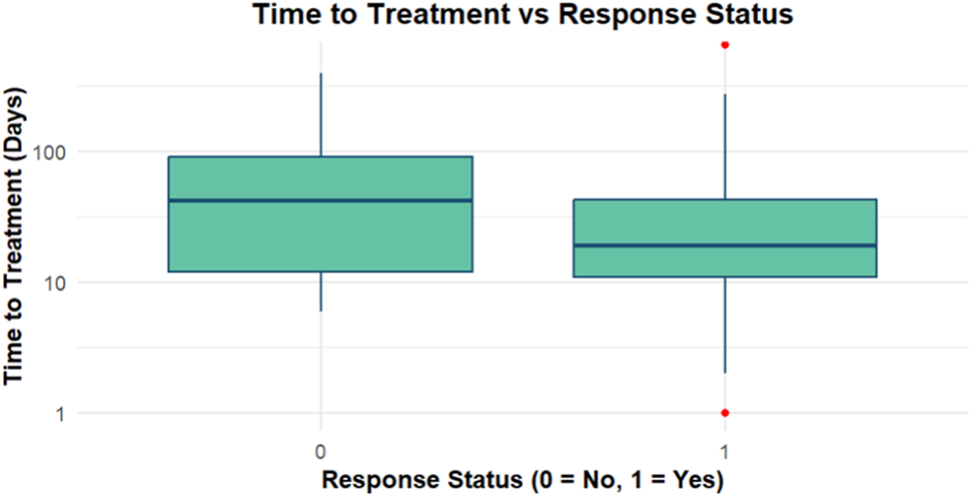
Box plot comparing the median time from symptom onset and treatment initiation in patients who had complete symptomatic resolution with any duration of antibiotic therapy, and those that did not have symptomatic resolution with antibiotic therapy.

**Results:**

Table 1 summarizes the characteristics of the study cohort. Definitive Lyme arthritis was diagnosed in 67 patients. Of these, 35 patients (52%) achieved complete symptom resolution after one course of antibiotics, and 45 patients (67%) achieved resolution after up to two courses. Doxycycline was used as first-line therapy in 97% of cases (Figure 1). Antibiotic-refractory Lyme arthritis (ARLA) developed in 20 patients (30%); of these, 7 required disease-modifying antirheumatic drugs (DMARDs), and 3 underwent synovectomy. ARLA was associated with incomplete response to a single antibiotic course and persistent joint effusion on post-treatment radiography (Table 2). Intra-articular steroid use prior to antibiotic therapy was associated with a threefold increased odds of ARLA, although this did not reach statistical significance. Patients who responded to antibiotics had a shorter median time to treatment (19 days) compared to non-responders (42 days) (Figure 2).

**Conclusion:**

In this cohort, 30% of patients were ultimately diagnosed with antibiotic-refractory Lyme arthritis (ARLA), a rate notably higher than the previously reported 10%. Despite advancements in Lyme disease diagnostics, we observed significant delays in diagnosis and initiation of appropriate antibiotic therapy, as well as the use of intra-articular steroids prior to antibiotics. These factors may contribute to the development of ARLA.

**Disclosures:**

All Authors: No reported disclosures

